# Campylobacters and their bacteriophages from chicken liver: The prospect for phage biocontrol

**DOI:** 10.1016/j.ijfoodmicro.2016.08.026

**Published:** 2016-11-21

**Authors:** Antung S. Firlieyanti, Phillippa L. Connerton, Ian F. Connerton

**Affiliations:** a Division of Food Sciences, School of Biosciences, University of Nottingham, Sutton Bonington Campus, Loughborough, Leicestershire LE12 5RD, United Kingdom; b Department of Food Science and Technology, Faculty of Agricultural Engineering and Technology, Bogor Agricultural University, Indonesia

**Keywords:** *Campylobacter*, Chicken, Liver, Bacteriophage, Food safety, Phage therapy

## Abstract

Consumption of foods containing chicken liver has been associated with *Campylobacter* enteritis. *Campylobacters* can contaminate the surface of livers post-mortem but can also arise through systemic infection of colonising bacteria in live birds. The use of bacteriophage to reduce levels of *Campylobacter* entering the food chain is a promising intervention approach but most phages have been isolated from chicken excreta. This study examined the incidence and contamination levels of *Campylobacter* and their bacteriophage in UK retail chicken liver. Using enrichment procedures, 87% of 109 chicken livers were surface contaminated with *Campylobacter* and 83% contaminated within internal tissues. Direct plating on selective agar allowed enumeration of viable bacteria from 43% of liver samples with counts ranging from 1.8–> 3.8 log_10_ CFU/cm^2^ for surface samples, and 3.0–> 3.8 log_10_ CFU/g for internal tissue samples. Three *C. jejuni* isolates recovered from internal liver tissues were assessed for their ability to colonise the intestines and extra-intestinal organs of broiler chickens following oral infection. All isolates efficiently colonised the chicken intestines but were variable in their abilities to colonise extra-intestinal organs. One isolate, CLB104, could be recovered by enrichment from the livers and kidneys of three of seven chickens. *Campylobacter* isolates remained viable within fresh livers stored at 4 °C over 72 h and frozen livers stored at − 20 °C over 7 days in atmospheric oxygen, and therefore constitute a risk to human health. Only three *Campylobacter*-specific bacteriophages were isolated, and these exhibited a limited host range against the *Camplylobacter* chicken liver isolates. All were identified as group III virulent bacteriophage based on their genome size of 140 kb. The application of broad host range group II virulent phages (8 log_10_ PFU/g) to liver homogenates containing *C. jejuni* strains of diverse origin at 4 °C resulted in modest but significant reductions in the viable counts ranging from 0.2 to 0.7 log_10_ CFU/g.

## Highlights

• A high proportion of retail chicken livers are contaminated with the pathogen *Campylobacter but few contain bacteriophage*
• *Campylobacter* contamination can be surface associated or internalised within chicken liver tissue
• *Campylobacters* and bacteriophage remain viable within fresh and frozen chicken livers
• Bacteriophages can reduce *Campylobacter* viable counts when applied to contaminated chicken liver homogenates
• *Campylobacter* liver isolates efficiently colonise the intestines of broiler chickens and can be recovered from livers

## 1 Introduction

Following emergence as an enteric pathogen in 1970s (Skirrow, 1977), *Campylobacter* has been a major concern worldwide. In the UK, *Campylobacter* is the most common bacterial cause of gastrointestinal infection recorded in the last two decades (Adak et al., 2005, Food Standards Agency, 2013). The total number of cases of *Campylobacter* infection during 2000–2012 was 781,581, from 1,052,581 laboratory confirmed cases of foodborne disease (Food Standards Agency, 2013). Campylobacteriosis is the most frequently reported foodborne disease but these figures belie actual unreported caseloads that are estimated to be 9 million and 1.3 million cases per year within the EU and USA, respectively (Centers for Disease Control and Prevention (CDC), 2014, EFSA, 2015).

The primary source of the major pathogenic species, *C. jejuni* and *C. coli*, are contaminated chicken and cattle meat (Adak et al., 2005, Suzuki and Yamamoto, 2009, Wilson et al., 2008), whereas less frequently they arise from wildlife (Hughes et al., 2009, Sippy et al., 2012), water, sewage and the environment (Jones, 2001, Waage et al., 1999). These bacteria are prevalent in offal, and in particular chicken liver (Cornelius et al., 2005, Kenar et al., 2009, Noormohamed and Fakhr, 2012, Noormohamed and Fakhr, 2013, Strachan et al., 2012, Vashin et al., 2009, Whyte et al., 2006). Dishes such as liver paté and liver parfait have been reported as potential transmission vehicles for outbreaks of foodborne disease (Centers for Disease Control and Prevention (CDC), 2013, Edwards et al., 2014, Hope et al., 2014, Inns et al., 2010, O'Leary et al., 2009, Wensley and Coole, 2013) and the number of cases is increasing (Little et al., 2010). Moreover, their presence could pose a risk to animal welfare as *Campylobacter* species have been associated with a disease affecting poultry liver termed vibrionic hepatitis (Crawshaw et al., 2015, Jennings et al., 2011, Stephens et al., 1998).

In some cases, the occurrence of *Campylobacter* in liver may be the result of contamination from the intestinal contents during processing (Barot et al., 1983). Nonetheless, isolation from the internal tissue of liver samples indicated that *Campylobacters* can be present in these organs (Cox et al., 2007). It has been recognised that bacteria can cross the intestinal barrier of animals and humans, a process known as bacterial translocation. In general, the lymphatic path is perceived as the more convincing primary route of the translocation as compared with the venous system (Balzan et al., 2007). In vitro studies have demonstrated that *Campylobacters* can translocate using either transcellular passage through the enterocytes or paracellular routes via the tight junctions (Backert et al., 2013). Specific translocation mechanisms have been elucidated for enteric pathogens such as *Salmonella*, which uses several routes to pass through the intestinal barrier to inhabit systemic organs (Watson and Holden, 2010). However, further studies are required to obtain evidence of the translocation mechanisms operating for *Campylobacters* in humans and animals (Backert et al., 2013). For example, the capacity of *C. jejuni* to colonise particular tissues is affected by the organism's ability to utilise specific nutrients - asparagine utilisation has been reported to improve the ability of the pathogen to colonise liver (Hofreuter et al., 2008).

Thorough cooking is the key to eliminating the risk of *Campylobacter* enteritis from poultry dishes. However, recipes for meals such as liver paté indicate minimal cooking to preserve the sensory properties and retain a pink appearance inside. To safely cook such dishes, critical core temperatures of 68–70 °C must be reached and held for periods as long as 45 min (Hutchison et al., 2015), which can result in unacceptable sensory characteristics (Whyte et al., 2006). Pre-cooking treatments could be applied to lower the initial contamination level, for instance by freezing and washing of the liver using organic acid (Harrison et al., 2013, Hutchison et al., 2015). However, the use of organic acid was found to cause a colour change or bleaching of the liver surface, and may not be effective for *Campylobacter* naturally present within the internal structures of the liver.

Bacteriophages have gained recognition as therapeutic agents to control pathogens in livestock and poultry (reviewed by Johnson et al., 2008), and represent a potential approach to control *Campylobacters* in livers**.** *Campylobacter* bacteriophages can be isolated from chicken meat and chicken excreta (Atterbury et al., 2003, Atterbury et al., 2005, El-Shibiny et al., 2005, Loc Carrillo et al., 2007) but to date attempts to isolate *Campylobacter* phages from chicken liver have not been reported. The application of a single dose or mixtures of *Campylobacter* phages have been reported to be effective in reducing the intestinal colonisation of chickens by *C. jejuni* and *C. coli* (El-Shibiny et al., 2009, Kittler et al., 2013, Loc Carrillo et al., 2005). The efficacy of the treatment varies depending on the phage type and dose, the phage-sensitivity of the host, the time interval post administration (Loc Carrillo et al., 2005) and the route of administration, i.e. by oral gavage or via chicken feed (Carvalho et al., 2010). Phage resistant *Campylobacter* have been reported post-treatment at relatively low frequencies of 2–4% (El-Shibiny et al., 2009, Hammerl et al., 2014, Loc Carrillo et al., 2005).

In this study, *Campylobacter* and their phages were isolated from retail chicken liver. *Campylobacter* isolates were tested for their ability to re-colonise extra-intestinal organs of chickens in order to identify *Campylobacter* isolates able to inhabit the liver of broiler chickens. Finally, virulent bacteriophages were applied to *Campylobacter* contaminated chicken liver homogenates to provide proof of principle that bacteriophages can reduce *Campylobacter* contamination within the liver matrix.

## 2 Material and methods

### 2.1 Bacterial strains and bacteriophage

*Campylobacter jejuni* PT14 (Brathwaite et al., 2013) was used as a reference strain and also for phage isolation and propagation. *Campylobacter jejuni* HPC5 (Loc Carrillo et al., 2005) and *C. jejuni* 81–176 (Korlath et al., 1985) were used as controls in the chicken colonisation experiments and the phage treatments of contaminated chicken livers. All *Campylobacter* isolates were cultured on blood agar base no. 2 CM0271 (Oxoid, Basingstoke, United Kingdom) supplemented with 5% defibrinated horse blood (TCS, Buckingham, United Kingdom) under microaerobic conditions (5% O2, 5% H2, 10% CO2, 80% N2) at 42 °C for 18–24 h. *Campylobacter* phages CP30A (GenBank accession number JX569801) and CPX (GenBank accession number JN132397) were propagated on *C. jejuni* PT14 or a contemporary *Campylobacter* isolate using the soft agar overlay method (Atterbury et al., 2003). Phages from the UK typing scheme (ɸ1 to ɸ16) were propagated as described by Frost et al. (1999). In order to obtain high titre stocks of bacteriophage, 30 ml volumes of plate lysates were centrifuged at 40,000*g* for 2 h at 4 °C. The pellets obtained were re-suspended in 1 ml of SM buffer (50 mM Tris·HCl (pH 7.5), 100 mM NaCl, 8 mM MgSO4, 0.01% Gelatin) to give a phage suspension containing approximately 10 log_10_ PFU/ml.

### 2.2 Preparation of chicken liver

Chicken liver samples were purchased from local supermarkets in Nottingham and Loughborough in the UK. Samples were kept at 4 °C and analysed before their expiry date as stated on the packaging. Each package contained 5–9 livers which were divided into two halves. Half of the liver was transferred into a stomacher bag (Seward Ltd., Worthing, UK) and 10 ml of Maximum Recovery Diluent CM733 (MRD; Oxoid; Basingstoke, UK) was added. The liver was gently massaged to re-suspend *Campylobacter* on the liver surface. To recover *Campylobacter* from internal tissues, the other half of liver was sterilised by dipping the liver into boiling water for 20–30 s (Whyte et al., 2006) and then tissue was excised with hot scalpel before being stomached with the addition of MRD (1:1 dilution ratio).

### 2.3 Isolation of *Campylobacter* from chicken liver

A 4 ml aliquot of suspension from the liver surface sample or the stomached internal tissue was transferred into 4 ml of enrichment media. This consisted of 2 × *Campylobacter* Enrichment Broth Lab135 (Lab M, Heywood, UK) made up with the addition of: 10% lysed horse blood (TCS), 0.25 g/l each of sodium pyruvate, sodium metabisulphite and ferrous sulphate (each from Sigma Aldrich, Poole, UK) and *Campylobacter* Enrichment Selectavial SV59 (Mast, Bootle UK), in a bijoux bottle. The total volume of 8 ml resulted in limited airspace in the bottle, hence maintaining microaerobic conditions during incubation at 37 °C for 48 h. Five 10 μl aliquots from each bijoux were dispensed onto mCCDA CM739 agar (Oxoid) prepared with the addition of *Campylobacter* selective supplement code (SR155, Oxoid) and additional Agar No. 1 (Oxoid) added to give 2% and then incubated at 42 °C for 48 h under microaerobic conditions. *Campylobacter* were confirmed after subculture, using microscopic observation of Gram stained cells, together with catalase and oxidase tests.

### 2.4 Enumeration of *Campylobacter*

*Campylobacter* was enumerated using the Miles and Misra technique, with serial dilutions prepared in MRD and 10 μl aliquots spotted in quintuplicate on 2% mCCDA before incubating under microaerobic conditions at 42 °C for 48 h. Typical *Campylobacter* colonies were counted and the total number calculated as either log_10_ CFU/g for internal tissue samples or log_10_ CFU/cm^2^ for surface liver samples.

### 2.5 Species identification and Fla-typing using PCR methodologies

*Campylobacter* DNA was isolated using the GenElute™ Bacterial Genomic DNA Kit according to manufacturer's instructions for Gram negative bacteria (Sigma-Aldrich, UK). The PCR methodology was based on conditions previously described by Linton et al. (1997) for species identification and by Elvers et al. (2008) for FlaA SVR-typing. The oligonucleotides were purchased from Eurofins (Ebersberg, Germany) and consisted of the primers HIP400F (5′-GAAGAGGGTTTGGGTGGTG-3′) and HIP1134R (5′-AGCTAGCTTCGCATAATAACTTG-3′) targeting the *C. jejuni* hippuricase gene, CC18F (5′-GGTATGATTTCTACAAAGCGAG-3′) and CC51R (5′-ATAAAAGACTATCGTCGCGTG-3′) specific for the *C. coli* aspartokinase gene, and FLA4R (5′-GGATTTCGTATTAACACAAATGGTGC-3′) and FLA625R (5′-CAAG[*AT*]CCTGTTCC[*AT*]ACTGAAG) for the *Campylobacter flaA* gene. To determine the Fla type of the *Campylobacter* isolates, the PCR products were purified using the Wizard® SV Gel and PCR Clean-Up System (Promega, Southampton, UK) and the DNA sequenced using the Eurofins MWG Value Read service.

### 2.6 Isolation and characterisation of bacteriophages from chicken liver

Bacterial lawns were prepared, plaque purified and the plaque forming units per ml (PFU/ml) were determined as previously described (Atterbury et al., 2003). The bacteriophages were diluted to the routine test dilution of approximately 6 log_10_ PFU/ml. Ten microliters of phage suspensions were dispensed onto the surface of the lawns of the test *Campylobacter* strain and then incubated under microaerobic conditions at 42 °C for 18–24 h. The lysis profiles of the isolates produced by each phage were scored according to the protocol described by Frost et al. (1999) for the UK phage typing scheme. Phage genomic DNAs were prepared as previously described (Loc Carrillo et al., 2007). PCR amplification of *Campylobacter* phage DNAs was performed using group III-specific primers CP853B (5′-TCGTTATACCACGGATATAG-3′) and CP854B (5′-TATAGGAGGGTTGTGAAATG-3′), the amplification products of which can discriminate CP30A and CP8-like bacteriophages (Siringan et al., 2014).

### 2.7 Colonisation of chickens with *Campylobacter*

Procedures for the chicken colonisation experiments were carried out as previously described (Loc Carrillo et al., 2005). For each *Campylobacter* isolate, a suspension of 7 log_10_ CFU was administered by oral gavage to seven 16-day-old broiler chickens (male Ross 308) reared under strict biosecure conditions. The birds were killed after 7 days, and the caecal content, liver, spleen, heart, kidney and breast meat were examined for the presence of *Campylobacter* by direct plating on mCCDA and by enrichment as describe in Section 2.3. These animal studies were conducted under the Animals Scientific Procedures Act (1986) and were approved by the University of Nottingham local ethical review committee.

1.1. Recovery and survival of *C. jejuni* in fresh and frozen chicken liver during storage

*Campylobacter*-free chicken livers were harvested from *Campylobacter*-negative broiler chickens reared under strict biosecure conditions. Fresh chicken livers were divided into sections weighing approximately 10 g. Each section was placed into a stomacher bag and weighed. The liver was then inoculated with 5 ml *C. jejuni* suspension containing 3 or 7 log_10_ CFU/ml, and replaced with sterile water for negative controls. The samples were stomached and stored at 4 °C for 72 h. Aliquots of 200 μl were taken for *Campylobacter* enumeration at 0, 8, 24, 32, 48, 56, and 72 h time intervals over the period. Frozen chicken liver was defrosted for 18 h at 4 °C prior to inoculation and storage at 4 °C or − 20 °C. The subsequent steps followed the same protocol as fresh liver samples but at daily intervals over 7 days. Three independent replicate experiments were performed with fresh and frozen livers.

### 2.8 Phage treatment of *Campylobacter* contaminated chicken livers

*Campylobacter*-free chicken livers (10 g) were stomached before the addition of *C. jejuni* suspensions to inoculum densities of approximately 3 log_10_ CFU/g (low inoculum) or 5 log_10_ CFU/g (high inoculum). The liver stomachates containing *C. jejuni* were treated with either a phage suspension at 8 log_10_ PFU/g or with an equivalent volume of SM buffer (mock treatment). *Campylobacters* were enumerated and the phage titred as indicated above, following incubation at 4 °C over 48 h. All experiments were performed in triplicate.

### 2.9 Statistical treatment of data

Statistical differences between paired control and treatment groups (using log_10_-transformed *Campylobacter* counts) were assessed by using the Student's *t*-test with significance p < 0.05. Differences between experimental groups were analysed by analysis of variance.

## 3 Results

### 3.1 Prevalence of *Campylobacter* in retail chicken liver

A total of 109 samples of retail chicken liver were analysed for the presence of *Campylobacter* recoverable from surface or internal tissues. Isolation was performed on 7 different batches within a 2 month period. There was a high prevalence of *Campylobacter* with 87.2% and 82.6% of samples positive from surface and inner tissues respectively (Table 1). Most samples contained low numbers of *Campylobacter* that were only recoverable by enrichment. Samples that could be enumerated contained *Campylobacter* in the range of 1.8–3.8 log_10_ CFU/cm^2^ for surface samples and 3.0–3.8 log_10_ CFU/g for internal tissue samples. Three surface samples and 5 internal tissue samples contained *Campylobacter* ≥ 3 log_10_ CFU/g, which would be considered to pose a significant risk to consumers (Food Safety Agency UK 2014).

### 3.2 Frequency and characteristics of *Campylobacter* phages in retail chicken liver

Three *Campylobacter*-specific bacteriophages were isolated from 109 retail chicken livers (2.7%). One of the phage originated from a surface sample (CLP6), while the other two were from the internal tissues of the livers (CLP47 and CLP63). Phages CLP47 and CLP63 exhibited similar lytic abilities against the *C. jejuni* liver isolates (64%), whilst phage CLP6 was virulent against more of the *C. jejuni* isolates (88%). However, the host ranges of the three new liver isolates were more specific than phages CP30A and CPX previously isolated from chicken intestinal contents or chicken meat respectively. None of the phage isolated from chicken liver infected the *C. coli* isolates.

*Campylobacter* bacteriophages possess double-stranded DNA genomes that are classified into three groups according to their genome size and head diameter, i.e. group I with genome sizes of 320 kb and head diameters of 140.6 and 143.8 nm; group II, with genome sizes of 184 kb and head diameters of 99 nm; and group III with genome sizes of 138 kb and head sizes of 100 nm (Sails et al., 1998). PFGE analysis of bacteriophage genomic DNA revealed that the three phages isolated from chicken liver were approximately 140 kb in size, which is typical of group III bacteriophages and similar to the reference phages CP30A and CPX. PCR amplification of the phage DNAs with group III-specific primers confirmed the classification of the liver phages.

### 3.3 Characterisation of *Campylobacter* isolates

A combination of Fla-typing and phage typing was used to discriminate the *Campylobacter* isolates, which enabled them to be placed into five groups that are summarised in Table 2. The *C. coli* isolates represent a single Fla-type that could not be distinguished with the phage used in this study. *C. jejuni* isolates could be placed in four groups where concordance was observed between the Fla-types and the phage sensitivity profiles. One group, represented by isolate CLB104, were recovered exclusively from the internal tissues of retail chicken liver with counts ≥ 3 log_10_ CFU/g, and therefore represents a significant risk to human health. *C. jejuni* isolates CLB44, CLB68 and CLB104 that originated from the internal tissues of chicken livers were selected for further study.

1.2. Persistence of *C. jejuni* and phage in fresh and frozen chicken liver during storage

**Fig. 1:**
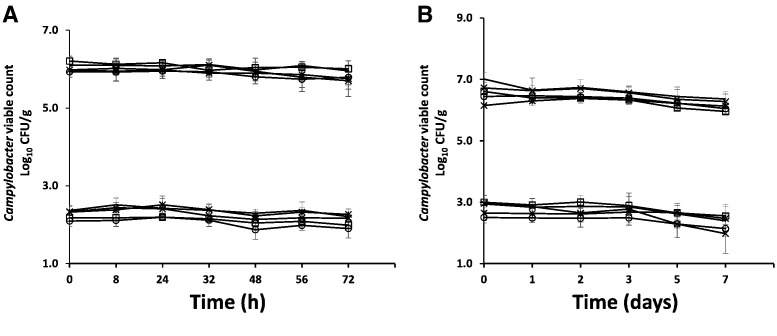
Viable *Campylobacter* counts in fresh and frozen chicken liver during storage: A) 72 h (fresh liver at 4 °C) and B) 7 days (frozen liver at − 20 °C) with either initial high (7 log_10_ CFU/g) or low (3 log_10_ CFU/g) target inoculums. Error bars represent the standard deviations for *n* = 3.

Microbiological analysis showed that the caecal contents, internal organs and breast meat of the experimental chickens were *Campylobacter*-free post-mortem prior to inoculation with test strains. Fresh and defrosted frozen livers were inoculated with 5 strains of *C. jejuni* at two inoculum levels of approximately 7 log_10_ CFU/g and 3 log_10_ CFU/g. The inocula were selected to represent the high and low contamination levels observed in this study. The recovery of *Campylobacter* counts from fresh and frozen livers immediately following inoculation was almost 100% demonstrating that the liver stomachates are not inimical to the survival of *Campylobacters*. Thereafter *C. jejuni* (control strains and liver isolates) remained viable at both inoculation levels throughout the storage period at 4 °C (Fig. 1). Mean reductions of 0.4–0.5 log_10_ CFU/g in the *Campylobacter* counts were observed for frozen liver samples. The greatest reduction recorded was 1.0 ± 0.74 log_10_ CFU/g from the low level inoculum of *C. jejuni* CLB44. In contrast, *C. jejuni* CLB104 showed no significant fall in the count under any circumstance (p > 0.05). All bacteriophage could be recovered from fresh or frozen liver stomachates without any significant fall in the inoculation titre of 8 log_10_ PFU/g over 72 h (p > 0.05).

### 3.4 Phage treatment of *Campylobacter* contaminated liver

**Fig. 2:**
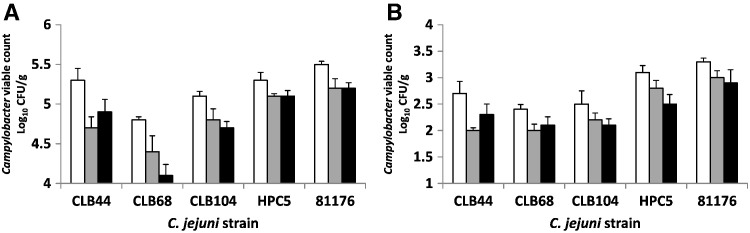
Phage activity against five *C. jejuni* strains in chicken liver: A) high contamination (5 log_10_ CFU/g) and B) low contamination levels (3 log_10_ CFU/g). *Campylobacter*-free liver samples were prepared by addition of *Campylobacter* suspensions after which, each was treated with either a phage suspension or with SM buffer (mock treatment). *Campylobacters* were enumerated following incubation at 4 °C for 48 h. White columns represent mock-treated samples, grey columns represent ɸ3 treatments and black columns represent ɸ15 treatments. Error bars represent the standard deviations for *n* = 3.

As noted above the bacteriophages isolated from liver have a restricted host range amongst the *Campylobacter* liver isolates compared to those of chicken intestinal (CP30A) or chicken meat (CPX) origin or the typing phages ɸ3 and ɸ15 (Table 2). Phages ɸ3 and ɸ15 have tailed morphologies and are classified as group II based on their genome sizes of 180 and 190 kb (Sails et al., 1998). However, ɸ3 and ɸ15 were able to lyse 3 of the 5 groups of *Campylobacter* liver isolates in addition to the control *C. jejuni* strains HPC5 (original source chicken intestine) and 81–176 (original source human with campylobacteriosis), and were therefore selected for phage therapy (biosanitization) applications with chicken liver to enable comparisons of the effect between *C. jejuni* strains. Phages ɸ3 or ɸ15 were added at 8 log_10_ PFU/g to liver stomachates containing either low (3 log_10_ CFU/g) or high (5 log_10_ CFU/g) target *Campylobacter* inoculums and stored at 4 °C over 48 h. Fig. 2 presents the viable counts of five *C. jejuni* strains following either mock or phage treatments of chicken liver suspensions. All the phage treated *C. jejuni* strains showed a significant reduction in the viable count compared to the control for low and high inoculums (p < 0.05). However, the reductions observed were modest. For example, the reductions in viable count recorded for the high risk livers represented by the high inoculum series of 5 log_10_ CFU/g ranged between 0.7 log_10_ CFU/g for the chicken liver isolate CLB68 treated with ɸ15 and 0.2 log_10_ CFU/g for the chicken intestinal isolate HPC5 treated with either ɸ3 or ɸ15. The phage recovered from these experiments showed minimal variation in titre and showed no significant difference to the initial inoculum titre (p > 0.05).

### 3.5 Capability of *Campylobacter* isolates to colonise broiler chickens

Cell suspensions of five *C. jejuni* cultures in physiological phosphate buffered saline (approximately 7 log_10_ CFU/ml) were administered orally, to 6 or 7 broiler chickens and colonisation was established after 7 days by examining post-mortem *Campylobacter* counts from chicken caecal contents and from extra-intestinal organs, i.e. liver, heart, spleen, breast muscle and kidney. There was no observable pathology for any of the organs. All chickens contained high counts of *Campylobacter* in their caecal contents (> 7 log_10_ CFU/g) based on enumeration on mCCDA plates (Table 3). However, *Campylobacters* could only be recovered from the extra-intestinal organs of chickens colonised by the liver isolates, and only by enrichment. *C. jejuni* isolate CLB104 was detected in the liver and kidney 3 of 7 chickens, while being recovered from all of the extra-intestinal organs of one bird. No *Campylobacters* were recovered from the excised breast meat of any chicken. No *Campylobacters* were recovered from the extra-intestinal organs of the control *C. jejuni* strains HPC5 or 81–176.

## 4 Discussion

*Campylobacter* was found in the majority of retail chicken liver samples at varying levels of contamination. Of concern are chicken meat samples containing > 3.0 log CFU/g, which pose a disproportionally high risk to consumer health (Food Standards Agency, 2015). However, we recorded *Campylobacter* counts > 3.0 log CFU/g for 2.8% of the surface and 4.6% of the internal tissue samples from retail chicken livers. A compilation of findings presented here with those available in the literature, are presented in Table 4, which demonstrates that *Campylobacter* contamination of livestock liver is prevalent with surveys recording that 66–100% of the samples tested were positive. In the majority of cases the livers showed a low level contamination, for example, Cornelius et al. (2005) and Whyte et al. (2006) found that 83–88% of internal tissues of livers harboured < 10^2^ MPN (most probable number) per g, while the remaining samples contained 10^2^–10^3^ MPN/g. A clear dose–response relationship between consumption of chicken liver paté and the risk of infection with *Campylobacter* has been demonstrated (Edwards et al., 2014). A low level of contamination does not eliminate the risk of *Campylobacter* infection since the infective dose can be as low as 500 cells (Robinson, 1981).

Whilst *Campylobacters* are frequently reported from liver, this is the first study to report the isolation of *Campylobacter*-specific bacteriophage. The isolation frequency was comparatively low at 2.7% but the phages recovered were generally able to infect the *Campylobacters* recovered from liver suggesting they are replicating in the source tissues. This would offer the prospect that phage therapy could be applied to control *Campylobacters* in vivo or on retail liver. The application of *Campylobacter*-specific bacteriophages has been demonstrated to successfully reduce contamination levels in chicken skin and meat (Atterbury et al., 2003, Bigwood et al., 2008, Goode et al., 2003). Similarly the application of phages ɸ3 and ɸ15 to chicken liver stomachates containing *C. jejuni* resulted in significant reductions in the viable counts of all five strains tested. However, the reductions observed post phage treatment in this study are unlikely to have a universal impact on the risk imparted by the consumption of chicken liver. As discussed above chicken meat containing > 3 log_10_ CFU/g represents a disproportionate risk, and viable count reductions in the range of 0.2 to 0.7 log_10_ CFU/g for the high level contamination series of 5 log_10_ CFU/g would not be sufficient to reduce the risk of infection. Whereas reductions of 0.7 log_10_ CFU/g in the viable count for lower levels of contamination, as demonstrated in the 3 log_10_ CFU/g series experiments in this study, could be of benefit. For the application to be of general use the levels of pathogen reduction need to be uniformly at the higher levels observed here, and the application would have to be on the liver before any processing for cooking and consumption. Bacteriophages in general have gained support for food sanitisation applications since bacteriophage capable of lysing the foodborne pathogens *Listeria monocytogenes* or *Salmonella* have been approved in the USA (US Food and Drug Administration and the Food Safety Inspection Service of the US Department of Agriculture) for use on retail food products.

The genome sizes of the chicken liver bacteriophages were estimated to be 140 kb using PFGE, which places them as group III *Campylobacter* bacteriophages (LLoc Carrillo et al., 2007, Sails et al., 1998). Recently a new sub-family of the T4-like phage super family, the *Eucampyvirinae*, has been proposed for *Campylobacter* bacteriophages based on their genomic DNA sequences/sizes and particle morphologies (Javed et al., 2014). Group III bacteriophages constitute the genus Cp8unalikeviruses with genome sizes in the range of 130–140 kb. The typing phages ɸ3 and ɸ15 used for phage therapy in this study are group II but also fall within the *Eucampyvirinae* as members of the genus CP220likeviruses. CP220likeviruses and Cp8unalikeviruses have been used successfully for active phage therapy in chickens against *Campylobacters* (El-Shibiny et al., 2009, Loc Carrillo et al., 2005, Hammerl et al., 2014, Scott et al., 2007), where there are sufficient densities of host bacteria to support phage replication (Cairns et al., 2009). Below the phage proliferation threshold requires that the bacteriophage encounter, adsorb and inundate the target bacteria - a process that has an intrinsic requirement for high phage titres. It is likely that some phages are better suited to this purpose in terms of achieving high titres, maintaining stability and retaining activity. In this application the phage titres applied to chicken liver would have to remain high at retail and post disruption of the liver when internalised bacteria may become accessible for phage lysis.

Details of the mechanisms involved in the intestinal colonisation of chickens are few but even less is known regarding how the liver may become colonised. Oral administration of the liver isolates to broiler chickens results in efficient intestinal colonisation but this does not guarantee liver colonisation. However, *C. jejuni* CLB104 could be recovered from the livers of some of the chickens, whereas the control *C. jejuni* strains remained within the intestine of all the birds to which they were administered. Jennings et al. (2011) examined the incidence of focal lesions in the livers of commercial broiler chickens as a characteristic of the disease vibrionic hepatitis. These authors noted that livers showing focal lesions were more likely to have greater *Campylobacter* content than those without but were unable to replicate the disease in healthy chickens inoculated with the liver isolates. However, that *Campylobacters* could colonise the livers of these chickens is of significance given the association of chicken liver with foodborne disease. More recently it has been reported that vibrionic hepatitis (spotty liver disease) in laying hens is associated with infection by a novel *Campylobacter* species exhibiting a new sub-lineage of 16S rRNA (Crawshaw et al., 2015). *Campylobacter jejuni* may not be the aetiological agent of vibrionic hepatitis in chickens but the ability of members of the species to cross the intestinal wall and reside in the liver in significant numbers within birds destined for retail represents another route of exposure to the consumer.

## Figures and Tables

**Table 1:** Prevalence and concentration of *Campylobacter* in retail chicken liver.

Sample	Samples containing *Campylobacter*	Samples with > 3.0 log CFU/cm^2^ or CFU/g	Number of *C. jejuni* isolates	Number of *C. coli* isolates
Liver surface	95/109 (87.2%)	3/109 (2.8%)	25/95 (26.3%)	70/95 (73.7%)
Internal tissue	90/109 (82.6)	5/109 (4.6%)	37/87 (42.5%)	50/87 (57.5%)

**Table 2:** Characteristics of *Campylobacters* isolated from livers.

Isolates	Species	Fla-Type	Bacteriophage lytic spectra^a^						
			CLP6	CLP47	CLP63	CP30A	CPX	ɸ3	ɸ15
CLB44	*C. jejuni*	32	OL	OL	OL	OL	OL	OL	OL
CLB56	*C. coli*	16	–	–	–	–	–	–	–
CLB62	*C. jejuni*	100	–	–	–	++	< SCL	–	–
CLB68	*C. jejuni*	16	< CL	–	–	< CL	–	+++	+++
CLB104	*C. jejuni*	18	–	–	–	–	–	SCL	+++

a Classification of phage infection based on that of Frost et al. (1999) using a routine test dilution of 6 log_10_ PFU/ml: CL confluent lysis; OL opaque lysis; SCL semi-confluent lysis; +++ > 100 plaques; ++ < 100 > 50 plaques; – no plaque formation.

**Table 3:** Recovery of *Campylobacter* from chicken intestines and extra-intestinal organs.

*Campylobacter* isolates	Caecal content (log_10_ CFU/g)	Recovery by enrichment				
		Liver	Heart	Spleen	Breast meat	Kidney
CLB44	7.7 ± 0.82	2/6 (33%)	1/6 (17%)	0/6 (0%)	0/6 (0%)	0/6 (0%)
CLB68	8.0 ± 0.37	1/7 (14%)	0/7 (0%)	0/7 (0%)	0/7 (0%)	1/7 (14%)
CLB104	7.5 ± 0.43	3/7 (43%)	1/7 (14%)	1/7 (14%)	0/7 (0%)	3/7 (43%)
HPC5	7.4 ± 0.70	0/7 (0%)	0/7 (0%)	0/7 (0%)	0/7 (0%)	0/7 (0%)
81–176	7.2 ± 0.47	0/7 (0%)	0/7 (0%)	0/7 (0%)	0/7 (0%)	0/7 (0%)

**Table 4:** Prevalence and number of *Campylobacter* in livestock and poultry liver.

References	Sample	Frequency	Samples contained > 3.0 log CFU/g
This study	Retail chicken liver surface	95/109 (87.2%)	3/109 (2.8%)
	Retail chicken liver internal tissue	90/109 (82.6)	5/109 (4.6%)
Harrison et al. (2013)	Chicken liver unfrozen	33/33 (100%)	30%
	Chicken liver frozen	30/30 (100%)	7%
Whyte et al. (2006)	Chicken liver surface	30/30 (100%)	30% (> 1.1 × 10^3^ MPN/sample)
	Chicken liver internal tissue	27/30 (90%)	6% (> 10^3^ MPN/g)
Noormohamed and Fakhr (2013)	Beef livers	39/50 (78%)	NA
Noormohamed and Fakhr (2012)	Chicken liver	122/159 (77%)	NA
Strachan et al. (2012)	Chicken liver	21/26 (81%)	25%
	Cattle liver	22/32 (69%)	25%
	Pig liver	23/29 (79%)	3%
	Sheep liver	31/40 (78%)	10%
Kenar et al. (2009)	Chicken liver surface	108/150 (72%)	NA
	Chicken liver internal tissue	30/150 (20%)	NA
Cornelius et al. (2005)	Sheep liver	180/272 (66%)	6.7% (> 10^2^ MPN/g or > 30 cells/g)
